# Immunohistochemical Expression of Haptoglobin in Skin Lesions of Hidradenitis Suppurativa

**DOI:** 10.3390/life15050738

**Published:** 2025-05-02

**Authors:** Nessr Abu Rached, Hanna Telkemeyer, Marina Skrygan, Martin Doerler, Yannik Haven, Lennart Ocker, Daniel Myszkowski, Thomas Meyer, Markus Stücker, Eggert Stockfleth, Falk G. Bechara

**Affiliations:** 1 International Centre for Hidradenitis Suppurativa/Acne Inversa (ICH), Department of Dermatology, Venereology and Allergology, Ruhr-University Bochum, 44791 Bochum, Germany; hanna.telkemeyer@kklbo.de (H.T.); marina.skrygan@klinikum-bochum.de (M.S.); martin.doerler@kklbo.de (M.D.); yannik.haven@kklbo.de (Y.H.); lennart.ocker@kklbo.de (L.O.); daniel.myszkowski@kklbo.de (D.M.); thomas.meyer@kklbo.de (T.M.); markus.stuecker@klinikum-bochum.de (M.S.); eggert.stockfleth@klinikum-bochum.de (E.S.); falk.bechara@kklbo.de (F.G.B.); 2 Skin Cancer Center, Department of Dermatology, Venereology and Allergology, Ruhr-University Bochum, 44791 Bochum, Germany

**Keywords:** hidradenitis suppurativa, HS, skin lesion, inflammation, meta-inflammation, haptoglobin, diabetes mellitus

## Abstract

Background: Meta-inflammation is a hallmark of hidradenitis suppurativa (HS). Research on meta-inflammation in HS is growing, but there is still no research on haptoglobin as an inflammatory protein in lesional HS skin. This study examines the relationship between haptoglobin expression in HS skin lesions and clinical parameters. Methods: An examination was performed on 44 skin samples from HS patients and 10 healthy skin samples. Clinical parameters were then compared with haptoglobin expression. Results: Median haptoglobin expression was significantly higher in the Hurley stage III lesions compared with milder stages (H-score: 37.6 versus 17.1, *p* = 0.028). High haptoglobin expression (≥30.8% positive cells) was associated with advanced disease (Hurley stage III: 80% versus 41.7%, *p* = 0.01), active smoking (80% versus 50%, *p* = 0.039), increased pain (visual analogue scale: 5 versus 1.5, *p* = 0.03), and a higher prevalence of diabetes (35% versus 8.3%, *p* = 0.029) and hypertension (55% versus 25%, *p* = 0.042). No significant associations were found with the BMI, disease duration, or CRP levels. Conclusions: High haptoglobin expression (positive cells ≥ 30.8%) in a skin lesion is associated with higher HS severity, active smoking, more pain and the comorbidities of diabetes mellitus and arterial hypertension in HS patients.

## 1. Introduction

Hidradenitis suppurativa (HS) is a chronic inflammatory skin disease characterised by painful, deep-seated nodules, abscesses, fistula tracts, and scarring in the inverse areas of the body [1,2,3]. HS is characterised by chronic meta-inflammation [4]. Meta-inflammation is a type of chronic, low-grade systemic inflammation that is closely associated with metabolic disorders [5]. This inflammation is associated with obesity, type 2 diabetes, and other metabolic diseases [6,7,8,9]. At the cellular level, macrophages, adipocytes, and other immune cells, among others, are involved in the process of meta-inflammation [10,11,12]. Optimal treatment for HS consists of multimodal therapy including surgical resection, antibiotics, psychological support, interdisciplinary management of comorbidities, local therapies, and biologicals [13]. Modification of the inflammatory response and inhibition of signalling pathways is an important therapeutic target in HS patients. Three biologics—adalimumab, secukinumab, and bimekizumab—are currently approved by the EMA for moderate-to-severe HS [14,15,16,17]. Other biologicals, such as JAK1 inhibitors or IL-17AF nanobodies, are expected to play an important role in the treatment of HS in the near future. However, target structures for HS therapies that belong to meta-inflammations are also increasingly being investigated. There is growing awareness that GLP-1 receptor agonists have significant potential as adjunctive treatments for HS, as they address both the metabolic and inflammatory aspects of the disease [18,19].

Similar to rheumatic diseases, several inflammatory markers are known to be associated with the severity of HS. Inflammatory markers that were previously investigated for their role in the severity of HS include C-reactive protein, the erythrocyte sedimentation rate, neutrophil-lymphocyte ratio, pan-immune inflammation score, and IgG [20,21,22,23,24]. In a previous analysis, we showed that circulating haptoglobin is a good marker of disease severity and metabolic risk in HS patients [25]. Haptoglobin is an acute-phase protein that has immunomodulatory properties and can therefore influence inflammatory responses. In addition, it acts as an antioxidant and plays a role in regulating the balance between type 1 and type 2 T-helper cells [26,27,28,29]. Haptoglobin activates the Th1 response [26,27]. HS is a predominantly Th1- and Th17-mediated immune response, and thus haptoglobin may play an important role via Th1 [30,31]. However, it is not known whether the inflammatory marker is also associated with HS severity in lesional skin. Haptoglobin expression in the lesional skin tissue of HS patients was never investigated. Therefore, the aim of this study was to fill this gap and analyse the influence of the inflammatory protein in HS skin.

## 2. Materials and Methods

### 2.1. Design, Sample Size, and Setting

Patient recruitment and sample collection took place at the International Centre for Hidradenitis Suppurativa/Acne inversa (ICH) at the University Hospital of the Ruhr University Bochum. Patient data and skin samples were obtained from a prospective study [25,32]. This study was conducted in accordance with the ethical principles of the Declaration of Helsinki. Clinical data and laboratory parameters were collected to document the participants’ demographic and health-related information. This included the age, sex, and body mass index (BMI). In addition, the duration of the disease and the severity of HS were recorded using the Hurley stages. Information on comorbidities such as obesity, diabetes mellitus, or cardiovascular disease was also collected.

Sample size calculation was not possible due to the lack of specific data on haptoglobin expression in HS in the literature. Therefore, an approximation was made based on comparable immunohistochemical studies of haptoglobin expression in psoriasis [33,34,35]. The sample sizes of comparable studies were 20–30 specimens for lesioned skin and 5–10 specimens for normal skin [33,34,35]. Therefore, skin tissue from 44 HS patients and 10 healthy controls was included in this study.

To retrospectively assess statistical significance, a power analysis was performed to compare haptoglobin H scores between Hurley III (n = 26) and Hurley I and II (n = 18). The calculated effect size was Cohen’s d = 1.02, which corresponds to a large effect. At a significance level of 0.05, the power achieved was 90.8%, confirming robust statistical power for this comparison. It can therefore be assumed that the differences observed in haptoglobin expression are methodologically reliable, despite the moderate sample size. The sample size was calculated using the post hoc power calculator available at https://clincalc.com/stats/power.aspx (accessed on 15 April 2025).

### 2.2. Haptoglobin Immunohistochemical Staining and Evaluation

Skin tissue from surgical excisions was used for this investigation. Formalin-fixed, paraffin-embedded skin tissue was cut into 4-µm-thick sections and transferred to microscope slides. Immunohistological staining of the skin tissue sections was carried out according to the protocol of Tian et al. using a mouse anti-human haptoglobin antibody (1:200 dilution; cat. no. ab13429; Abcam, Cambridge, UK) [36].

The slides were scanned using a Hamamatsu NanoZoomer S60 slide scanner (Hamamatsu City, Shizuoka Prefecture, Japan). The positively stained cells in the five fields of view were counted manually at 400× magnification. The counting was performed by two independent investigators. The two immunohistochemistry evaluators were blinded and had no clinical information. Immunohistochemical evaluation was performed in the inflammatory infiltrate of the dermis. For immunohistochemical analysis of haptoglobin, the H score and the absolute and relative number of positive cells were determined. The investigators categorised the colour intensity of the positive cells as weak, moderate, or strong staining. The H score was calculated using the following formula [37]:

H-score = (1 × percentage of weak staining) + (2 × percentage of moderate staining) + (3 × percentage of strong staining)

### 2.3. Statistical Analysis

Summary statistics will be generated for all variables collected, including patient demographics (e.g., age, sex, and BMI), disease-specific characteristics (e.g., Hurley stage, modified Hidradenitis Suppurativa Score [mHSS], Severity Assessment of Hidradenitis Suppurativa [SAHS] [38], and Dermatology Life Quality Index [DLQI] [39]) and co-morbidities (e.g., diabetes and hypertension). Continuous variables such as age and BMI are presented as medians with interquartile ranges. Binomial and categorical variables, including Hurley stages, are reported as frequencies and percentages. Comparative analyses were performed to evaluate the relationships between the haptoglobin expression levels and clinical variables. A chi-squared test was used for binary or categorical variables, and the Mann–Whitney U test was used for continuous variables. A *p* value of less than 0.05 was considered statistically significant for all tests.

## 3. Results

### 3.1. Personal and Clinical Characteristics of the HS Cohort

The present investigation comprised 44 patients diagnosed with HS (Table 1). Of these patients, 15 (34.1%) were female, and 29 (65.9%) were male, with a median age of 46.5 years (interquartile range (IQR): 33.8–57). The median age of disease onset was 27.5 years (IQR: 19–37.3). In addition, the median disease duration was recorded to be 11 years (IQR: 7–21.8). Further analysis revealed a median body mass index (BMI) of 32.0 kg/m^2^ (IQR: 29.3–36.8). A positive family history of HS was reported by 13 patients (29.6%), while 31 patients (70.4%) reported no family history. With respect to smoking habits, 28 patients (63.6%) were current smokers, 5 (11.4%) were ex-smokers, and 11 (25%) were non-smokers. The median modified Hidradenitis Suppurativa Score (mHSS) was 52.5 (IQR: 24.8–89.3). The median Severity Assessment of Hidradenitis Suppurativa (SAHS) score was 8 (IQR: 6–9), and the median Dermatology Life Quality Index (DLQI) score was 14 (IQR: 10–20).

### 3.2. Immunohistochemical Expression of Haptoglobin in Lesional Skin from HS Group and Healthy Skin from Controls

**Figure 1:**
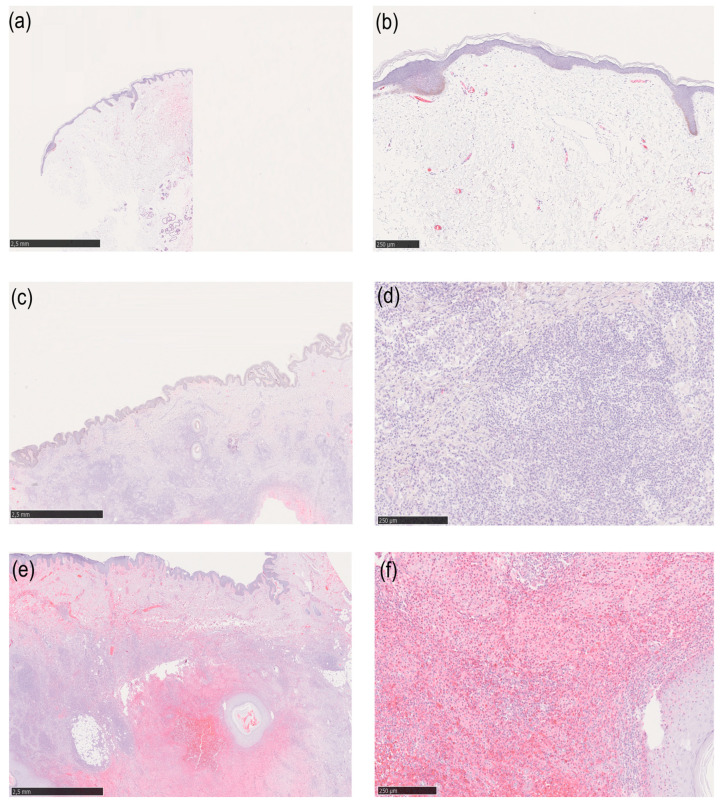
All figures show immunohistochemical staining with haptoglobin. (**a**) Overview of healthy skin. (**b**) Healthy skin at 100× magnification. (**c**) Overview of lesional HS skin with little haptoglobin expression. (**d**) Lesional HS skin with little haptoglobin expression at 100× magnification. (**e**) Lesional HS skin with high haptoglobin expression at overview. (**f**) Lesional HS skin with high haptoglobin expression at 100× magnification.

Haptoglobin expression was analysed and compared between skin lesions of patients with hidradenitis suppurativa (HS) and healthy controls, as well as between different anatomical regions affected by HS (Table 2 and Table 3). As expected, no significant differences in age and sex were observed between HS patients (n = 44) and controls (n = 10), since the controls were matched for age and sex (*p* > 0.05). HS lesions had significantly higher H-scores (median 31.2 versus 0.02; *p* < 0.001; Figure 1), absolute numbers of haptoglobin-positive cells (median: 245.8 versus 1; *p* < 0.001), and percentages of positive cells (median: 26.8% versus 0.02%; *p* < 0.001). Further analysis revealed differences in haptoglobin expression between different regions affected by HS. Axillary involvement showed the highest median H score (35.4), absolute number of positive cells (361.8), and percentage of positive cells (32.0%), followed by perianal involvement with a median H score of 31.7, 288.5 positive cells, and 29.7% positive cells. In contrast, the lowest expression levels were observed in the mons pubis region, where the median H score was 24.8, the absolute number of positive cells was 191.1, and the percentage of positive cells was 18.5%.

### 3.3. Clinical HS Characteristics and Haptoglobin Expression

Patients with Hurley stage III disease had significantly higher H scores (median: 37.6 versus 17.1, *p* = 0.028; Table 4), a greater absolute number of positive haptoglobin-expressing cells (median: 373.1 versus 187.6, *p* = 0.048), and a trend towards a higher percentage of positive cells (median: 34.8% versus 18.4%, *p* = 0.059). Elevated CRP levels were associated with a higher absolute number of positive cells (median: 390.5 versus 174.7, *p* = 0.04), and there was a borderline significant increase in the H scores (median: 40.5 versus 17.1, *p* = 0.053). Patients with hypothyroidism had significantly higher H scores (median: 61.5 versus 29.1, *p* = 0.045), a greater absolute number of positive cells (median: 549.8 versus 234, *p* = 0.03), and a higher percentage of positive cells (median: 55.4% versus 30.8%, *p* = 0.045). Other parameters, including sex, family history of HS, diabetes, hypertension, obesity, and CRP levels, did not show significant differences in their H scores, absolute positive cell counts, or percentage of positive cells, except for the trends observed in patients with diabetes (*p* = 0.078) and hypertension (*p* = 0.055).

### 3.4. Comparison of Clinical Characteristics at Low and High Haptoglobin Expression

To determine the clinical differences between low and high haptoglobin expression, we performed an ROC analysis. Our investigations showed that both serum and skin tissue haptoglobin were related to the severity of HS. In the ROC analysis, the percentage of positive area for haptoglobin was used as the test variable, and Hurley III was used as the state variable. With a Youden index of 0.393, a cut-off value of 30.8 was determined. Table 5 compares the clinical characteristics between the patients with low (<30.8%) and high (≥30.8%) haptoglobin expression in HS. A significantly higher proportion of patients with high haptoglobin expression was active tobacco smokers (80% versus 50%, *p* = 0.039). SAHS was also significantly higher in this group (median: 8.5 versus 7, *p* = 0.008). Advanced disease, as indicated by Hurley stage III, was more common in patients with high haptoglobin expression (80% versus 41.7%, *p* = 0.01). Patients with high haptoglobin expression also reported higher visual analogue scale (VAS) scores for current pain (median: 5 versus 1.5, *p* = 0.03) and had a higher prevalence of diabetes mellitus (35% versus 8.3%, *p* = 0.029) and hypertension (55% versus 25%, *p* = 0.042). Blood haptoglobin levels were significantly elevated in patients with high expression (median: 227.3 versus 188.6, *p* = 0.016). However, there were no statistically significant differences between the two groups in terms of age, age at disease onset, disease duration, BMI, family history of HS, number of flares in the previous 4 weeks, or CRP levels.

## 4. Discussion

This investigation provides new and important insights into the role of haptoglobin in the inflammatory process of HS. Serum haptoglobin level is an independent marker of HS disease severity and metabolic risk [25,40]. The findings of this study demonstrate that elevated levels of haptoglobin expression in HS skin lesions (defined as ≥30.8% positive cells) are associated with increased disease severity and a greater prevalence of comorbidity. In particular, patients with Hurley stage III disease were found to have significantly higher haptoglobin expression than patients with milder disease. This highlights the potential role of haptoglobin as a marker of disease severity [25]. Activation of the Th1 and Th17 immune response is characteristic of HS. This inflammatory pathway could be enhanced by increased skin tissue haptoglobin, as haptoglobin leads to promotion of the Th1 response and modulation of oxidative stress [41,42,43,44,45]. Haptoglobin functions as a regulatory agent within the Th1/Th2 balance [27]. An association between haptoglobin in skin tissue and disease severity was also found in other chronic diseases. A study by Lee et al. showed similar results for chronic obstructive pulmonary disease (COPD), which is caused by chronic inflammation [46]. Haptoglobin levels influence the severity of COPD [46]. Another study analyzed haptoglobin in urine and found that this biomarker can predict mortality risk in patients with type 2 diabetes independent of traditional risk factors [47].

Furthermore, the analysis shows that high haptoglobin expression is associated with an increased risk of metabolic comorbidities such as diabetes mellitus and arterial hypertension. This association may be explained by the inflammation-modulating properties of haptoglobin, which were identified in previous studies as contributing to systemic inflammation and metabolic dysregulation. Higher haptoglobin concentrations and more meta-inflammation are associated with poorer blood sugar control, showing a significantly increased level of fasting blood sugar [40].

The connection between HS and haptoglobin can also be readily elucidated at the cellular level. Haptoglobin is considered an acute-phase protein and is produced primarily in hepatocytes in the liver. The cytokines IL-6, TNF-α, and IL-1β bind to their respective receptors on the surface of hepatocytes and activate signalling cascades that promote the expression of haptoglobin [48,49,50,51,52,53,54,55]. Haptoglobin is released by a variety of cells, including macrophages, endothelial cells, fibroblasts, and neutrophils [29,56,57,58,59]. T cells also contribute indirectly to the secretion of haptoglobin by promoting the activation of macrophages [60]. All three cytokines—IL-6, TNF-α, and IL-1β—are elevated in HS patients and could explain the increased haptoglobin levels in skin tissue and serum [61,62,63].

Genetic predisposition to haptoglobin genotypes also plays an important role in inflammation. The HP2 allele and HP 2-2 genotype are associated with several immune disorders, such as inflammatory bowel disease and systemic lupus erythematosus [64,65,66]. A similar association was found in HS. Genotype haptoglobin 2-2 is associated with familial hidradenitis suppurativa and confirms that the allele Hp2 is a predisposing factor for autoimmune inflammatory diseases [32].

Our results show that patients with high haptoglobin expression reported significantly greater pain. This could be explained by increased activation of pro-inflammatory pathways that promote both peripheral and central sensitisation [67,68]. In addition, the link between more pain and more local haptoglobin may reflect the greater activity of acute inflammation.

HS patients with high haptoglobin expression also reported higher pain intensities, and a higher proportion were active smokers. Furthermore, the link between high haptoglobin expression and active smoking suggests that exogenous factors may influence inflammation in skin tissue. Smoking is a known trigger of an increased systemic inflammatory response, which may explain why patients with high haptoglobin expression were also more likely to be active smokers [69,70,71].

Consequently, the results of this study could have significant clinical implications. On the one hand, the correlation between elevated levels of haptoglobin expression in lesional skin and more severe HS, higher pain intensities, and a greater prevalence of comorbidities, including diabetes mellitus and arterial hypertension, suggests that skin tissue haptoglobin could serve as a biomarker for patient stratification. This would facilitate the early identification of patients who are at higher risk of severe disease progression or systemic involvement, thereby enabling the adjustment of monitoring and therapeutic measures accordingly. Moreover, the correlation between haptoglobin expression and inflammatory and metabolic parameters suggests that haptoglobin may be a useful indicator in the selection of therapeutic interventions. Patients exhibiting elevated local or systemic haptoglobin expressions may therefore benefit from anti-inflammatory or metabolically effective therapeutic approaches, such as GLP-1 receptor agonists. Finally, longitudinal measurement of skin tissue haptoglobin expression has the potential to facilitate prognosis assessments or evaluate therapy success, thereby contributing to more personalised management of HS. However, it is first necessary to conduct longitudinal studies on haptoglobin in skin tissue.

Nevertheless, the results of this study have to be interpreted with some limitations. The small sample size may limit the generalisability of the results, and the cross-sectional analysis does not allow causal conclusions. Further research with larger cohorts and longitudinal designs is needed to confirm the findings and gain a better understanding of the underlying mechanisms.

## 5. Conclusions

Haptoglobin in skin tissue is involved in the inflammatory process of HS patients. High haptoglobin expression (positive cells ≥ 30.8%) in skin lesions is associated with higher HS severity, active smoking, more pain, and the comorbidities of diabetes mellitus and arterial hypertension in HS patients.

## Figures and Tables

**Table 1:** Personal and clinical characteristics of 44 HS patients.

Parameter		Value (s)
Sex (n) (%)	female	15 (34.1%)
	male	29 (65.9%)
Age, median (IQR) (y)		46.5 (33.8–57)
Age of onset, median (IQR) (y)		27.5 (19–37.3)
Disease duration, median (IQR) (y)		11 (7–21.8)
BMI, median (IQR) (kg/m^2^)		32.0 (29.3–36.8)
Family history of HS, n (%)	positive	13 (29.6)
	negative	31 (70.4)
Tobacco smoker (n) (%)	current smoker	28 (63.6%)
	ex-smokers	5 (11.4%)
	non-smoker	11 (25%)
mHSS, median (IQR)		52.5 (24.8–89.3)
SAHS, median (IQR)		8 (6–9)
DLQI, median (IQR)		14 (10–20)

Note: n = absolute number of patients; SD = standard deviation; y = years; IQR = interquartile range; BMI = body mass index; HS = Hidradenitis suppurativa; mHSS = modified Hidradenitis Suppurativa Score; SAHS = Severity Assessment of Hidradenitis Suppurativa; DLQI = Dermatology Life Quality Index.

**Table 2:** Comparison of haptoglobin expression in skin between skin lesions from HS patients (n = 44) and healthy skin from controls (n = 10), compared using Mann–Whitney U and chi-squared tests.

Parameter	Hidradenitis Suppurativa (n = 44)	Controls (n = 10)	*p* Value
Male (n) (%)	29 (65.9%)	6 (60.0%)	0.7
Age, median (IQR) (y)	46.5 (33.8–57)	42.5 (45.5–55)	0.4
H score, median (IQR)	31.2 (10.0–48.7)	0.02 (0–0.03)	<0.001 *
Absolute number of positive cells for haptoglobin, median (IQR)	245.8 (46.7–429.1)	1 (0–1)	<0.001 *
Positive cells for haptoglobin (%)	26.8 (6.6–47.0)	0.02 (0–0.03)	<0.001 *

Note: n = absolute number of patients; y = years; IQR = interquantile range; * = significant result.

**Table 3:** Haptoglobin expression according to the pattern of involvement in HS.

Parameter	H Score ^1^	Absolute Number of Positive Cells for Haptoglobin ^1^	Positive Cells for Haptoglobin (%) ^1^
Axillary HS involvement	35.4 (18.8–49.3)	361.8 (151.8–455.3)	32.0 (14.4–47.4)
Mons pubis HS involvement	24.8 (7.2–46.3)	191.1 (39–433.3)	18.5 (6.0–43.0)
Inguinal HS involvement	27.5 (3.7–48.6)	209.6 (47.2–423.8)	23.0 (3.8–46.6)
Genital HS involvement	32.6 (15.1–46.4)	253.0 (88.8–425.0)	25.4 (12.4–45.1)
Perianal HS involvement	31.7 (17.8–51.2)	288.5 (158.9–433.3)	29.7 (17.1–48.8)
Gluteal HS involvement	31.5 (1.6–44.2)	318.6 (16–423.8)	29.6 (1.3–43.6)

^1^ Median (interquantile range).

**Table 4:** Comparison of clinical HS characteristics and haptoglobin expression in lesional HS skin, compared using Mann–Whitney U test.

Parameter	H-Score	*p* Value	Absolute Number of Positive Cells for Haptoglobin	*p* Value	Positive Cells for Haptoglobin (%)	*p* Value
Hurley III vs. Hurley II and I	37.6 (25.5–49.8), 17.1 (1.5–30.9)	0.028 *	373.1 (171.9–444.4), 187.6 (14.4–298.6)	0.048 *	34.8 (18.4–48.9), 18.4 (1.4–29.2)	0.059
Male vs. Female	31.0 (4.1–45.6), 35.8 (24.8–54.6)	0.2	253 (36.0–428.2), 238.6 (160.5–468.9)	0.5	25.4 (4.1–43.6), 28.3 (18.5–53.7)	0.3
CRP increased vs. not increased *	40.5 (26.8–49.4), 17.1 (4.0–32.6)	0.053	390.5 (192.0–452.0), 174.7 (38.3–320.0)	0.04 *	37.7 (20.8–48.4), 14.4 (4.0–30.2)	0.066
Patients with positive family history vs. without	24.9 (4.1–45.6), 31.5 (14.8–48.9)	0.5	253 (33–421.8), 238.6 (92.4–440.2)	0.6	28.3 (9.2–47.9), 25.4 (4.1–43.6)	0.7
Patients with diabetes vs. without	46.2 (35.9–59.0), 27.5 (6.2–44.9)	0.078	428.2 (324–448.6), 209.6 (42.5–411.1)	0.1	46.4 (33.8–55.4), 21.7 (5.4–42.7)	0.1
Patients with hypertension vs. without	45.6 (24.9–59.0), 27.5 (6.2–37.5)	0.055	364.6(192.8–448.6), 191.8 (42.5–401.7)	0.1	43.6 (21.5–55.4), 19.7 (5.4–34.7)	0.052
Patients with obesity vs. without	30.9 (7.2–47.0), 31.2 (17.5–49.0)	0.6	215.7 (39–424.9), 341.6 (176.5–461.3)	0.3	23.5 (6.0–47.1), 30.8 (17.9–47.0)	0.6
Patients with hypothyroidism vs. without	61.5 (34.2–62.6), 29.1 (8.3–44.2)	0.045 *	549.8 (310–574.9), 234 (40.0–400.4)	0.03 *	55.4 (33.2–61.2), 30.8 (6.6–41.7)	0.045 *

CRP = C-reactive protein (reference for CRP level < 5 mg/L); * = significant result.

**Table 5:** Comparison of clinical characteristics at low (positive amount for haptoglobin (%) < 30.8%) and high (positive amount for haptoglobin (%) ≥ 30.8%) haptoglobin expression compared using Mann–Whitney U and chi-squared tests.

Parameter	Positive Amount for Haptoglobin (%) < 30.8% (n = 24)	Positive Amount for Haptoglobin (%) ≥ 30.8% (n = 20)	*p* Value
Male (n) (%)	16 (66.7)	13 (65.0)	0.9
Age, median (IQR) (y)	40.5 (34–55.3)	55.5 (33.8–57.8)	0.1
Age of onset, median (IQR) (y)	28 (18.8–38.5)	26 (20–36.3)	0.9
Disease duration, median (IQR) (y)	10 (5.8–13.5)	15 (7.8–28.3)	0.1
BMI, median (IQR) (kg/m^2^)	31.7 (26.5–35.0)	35.9 (29.4–37.6)	0.1
Family history of HS (n) (%)	7 (29.2)	6 (30.0)	>0.9
Active tabacco smoker (n) (%)	12 (50.0)	16 (80.0)	0.039 *
mHSS, median (IQR)	31.5 (20.3–77.8)	71.5 (48–93)	0.06
SAHS, median (IQR)	7 (5–8)	8.5 (7–11)	0.008 *
DLQI, median (IQR)	13 (5.5–17)	16 (13.3–23.3)	0.09
Hurley III (n) (%)	10 (41.7)	16 (80.0)	0.01 *
Number of HS flare-ups in the last 4 weeks, median (IQR)	0 (0–1.3)	0.5 (0–2.5)	0.4
Current pain on VAS, median (IQR)	1.5 (0–3.3)	5 (1.5–7)	0.03 *
Diabetes mellitus (n) (%)	2 (8.3)	7 (35.0)	0.029 *
Obesity (n) (%)	16 (66.7)	12 (60.0)	0.6
Hypothyroidism (n) (%)	2 (8.3)	5 (25.0)	0.1
Hypertension (n) (%)	6 (25.0)	11 (55.0)	0.042 *
Leukocyte count in blood, median (IQR)	9685 (7255–11,863)	10,050 (7893–11,780)	0.7
CRP level in blood, median (IQR)	5 (5–11.1)	8.6 (5.3–17.0)	0.077
Haptoglobin level in blood, median (IQR)	188.6 (120–213.7)	227.3 (200.5–283.7)	0.016 *

VAS = visual analogue scale; n = absolute number of patients; y = years; IQR = interquartile range; BMI = body mass index; HS = Hidradenitis suppurativa; mHSS = modified Hidradenitis Suppurativa Score; SAHS = Severity Assessment of Hidradenitis Suppurativa; DLQI = Dermatology Life Quality Index; CRP = C-reactive protein; * = significant result.

## Data Availability

Data are contained within the article.
